# Differences in anatomoclinical profiles between RAS-Mutated and Wild-Type colorectal cancers in an Eastern Algerian Cohort

**DOI:** 10.4314/ahs.v26i1.12

**Published:** 2026-03

**Authors:** Yahia Massinissa, Afaf Benhouda, Hachani Khadraoui, Djahida Benouda, Wafa Khettache, Atika Soussi

**Affiliations:** 1 Biotechnology's Laboratory of the Bioactive Molecules and the Cellular Physiopathology, Faculty of Biological Sciences, Department of Biology and Living Organisms, University of Mustapha Ben Boulaid. 53 Constantine Rd, Fésdis, Batna 05078, Algeria. Tel: +213-676- 002083; 2 Laboratory of Anapathology, CHU of Batna, Algeria; 3 Department of Molecular and Cellular Biology, University of Khenchela Abbes Laghrour. 40000

**Keywords:** CCR, mutated KRAS, wild-type KRAS, mutations, Algeria

## Abstract

**Background:**

Colorectal cancer (CRC) ranks as the third most prevalent malignancy globally, with incidence rates rising steadily in Algeria. Prognostic assessment of CRC increasingly relies on molecular profiling, particularly mutations in the KRAS gene a critical determinant of resistance to anti-EGFR therapies in metastatic disease.

**Objectives:**

This study investigates the KRAS mutational spectrum and its association with clinicopathological features in CRC patients from Batna, Eastern Algeria.

**Methods:**

In a retrospective analysis of 91 cases, KRAS mutations were identified in 46.2% of tumors (wild-type: 58.3%) using RT-PCR and sequencing.

**Results:**

The most frequent alterations localized to codons 12 and 13 (exons 1–2), with p.G12D (c.35G>A) and p.G13D (c.38G>A) predominating. Strikingly, KRAS mutation status showed no significant correlation with age, sex, tumor size, histology, metastatic pattern, or TNM stage suggesting its role as an independent molecular driver rather than a surrogate for conventional prognostic markers.

**Conclusion:**

These findings underscore the high prevalence of KRAS mutations in Algerian CRC patients and highlight their potential utility in refining therapeutic strategies, particularly for anti-EGFR eligibility. The study provides the first regional dataset from Eastern Algeria, addressing a critical gap in North African oncogenomics.

## Introduction

Colorectal cancer (CRC) is a leading cause of cancer-related morbidity and mortality worldwide, with a rising incidence in developing nations^36^. Alarmingly, 20–25% of patients already present with metastatic disease at diagnosis, and 75–90% of these metastases evade early detection, drastically limiting curative options^16^. In Algeria, CRC represents a critical public health crisis, constituting nearly 50% of digestive cancers—which themselves account for 20–25% of all malignancies nationwide^2^. The disease exhibits a male predominance, with most cases emerging after age 50, positioning CRC as the second most diagnosed cancer and a leading cause of cancer deaths in the country.

The pathogenesis of CRC follows a stepwise progression from adenomatous polyps to invasive adenocarcinoma, fueled by cumulative genetic and epigenetic disruptions^6^. Early carcinogenesis is often triggered by APC gene inactivation, leading to dysregulated β-catenin signaling in 70–80% of sporadic CRC cases^17^. Equally pivotal are activating mutations in KRAS, an oncogenic driver present in 30–50% of CRCs, which not only sustain tumor proliferation but also confer resistance to anti-EGFR therapies—a major therapeutic hurdle in metastatic disease^1^,^38^. Beyond genetic mutations, epigenetic mechanisms, such as CpG island hypermethylation, further silence tumor suppressors (e.g., MLH1, CDKN2A), exacerbating malignant progression^9^.

Despite advances in molecular profiling, regional disparities in CRC biology remain understudied, particularly in North Africa. While KRAS mutations are well characterized in Western and Asian cohorts, their clinicopathological implications in African populations—notably Algerians—are poorly defined^4^. Given Algeria's unique genetic and environmental risk factors (e.g., dietary habits, consanguinity rates), regional studies are imperative to optimize precision oncology strategies.

This study investigates the KRAS mutational landscape in an Eastern Algerian CRC cohort, correlating genetic profiles with clinicopathological features (e.g., tumor location, stage, histology) and prognostic outcomes. By addressing this gap, we aim to enhance risk stratification and inform therapeutic decision-making in a historically underrepresented population. Our findings could pave the way for tailored biomarker-driven therapies, ultimately improving CRC management in North Africa.

## Materials and methods

### Patient presentation

This is a retrospective, descriptive study performed on 91 patients (46 women and 45 men) aged 23 to 84 years with a CRC diagnosed between 2011 and 2019 at the anatomy and pathology laboratory and oncology at the Batna Anti-Cancer Center.

### Inclusion and exclusion criteria Inclusion criteria

- Having been admitted and followed to the oncology department level of BATNA CHU and the BATNA Anti-Cancer Center
- Both sexes (men and women) are included in this study.
- Having the KRAS analysis results.

### Exclusion criteria

- All patients who achieved CRC were excluded without KRAS analysis.

### Localization of the primary tumor

We searched for the location of the tumor in the right colon, left colon, rectum, sigmoid, appendix, Caecum, right colic angle, recto-sigmoid, recto-sigmoid hinge, colectomy, anal canal, plural, ileocolic.

### Macroscopic appearance

Naked eye observation of tissue alterations with a description of the lesion observed, this observation is made after a given organ removal or portion (part) of the organ placed in 10% formalin and is performed by the doctor of anatomopathological.

### Type of metastasis

We looked for different metastases such as: pulmonary, hepatic, peritoneal, bone, umbilical, ovarian, adrenal, intestinal, muscle, appendix, duodenum, rectal peritoneal, mediatalis with peritoneal carcinomatosis, multiple, alone.

### Analysis of mutations of the RAS gene

The analysis is performed in the laboratories of BioMarker Solutions Ltd London, UK and GENEKOR Committed to Biotechnological Innovation, and Oncopharmacology Laboratory, Center for Cancer Control, Nice France and London Laboratory for Molecular Biology and Cytogenetics.

The goal of the analysis is to detect patients with metastatic colorectal cancer in patients who are on ineffective anti-EGFR therapy due to the presence of codon 12, 13, 59, 61, 117, and 146 mutations. KRAS and NRAS genes. It will also be used to evaluate the BRAF V600E mutation as a prognostic marker^10^.

The applicant uses the TruSight™ Tumor 15 commercial kit (TST-15) and Illumina's MiSeq™ platform to perform the SNG. This kit is designed to perform multiplex PCR from DNA taken from formaldehyde-fixed, paraffin-embedded tissue samples. The TST-15 kit provides amplicon coverage of specific regions of 15 cancer-related genes, including the three genes targeted by this assay: AKT1, BRAF, EGFR, ERBB2, FOXL2, GNA11, GNAQ, KIT, KRAS, MET, NRAS, PDGFRA, PIK3CA, RET, TP53. Detection of mutations whose allele frequency is greater than 5% with a minimum coverage of 500 X can be performed by the TST-15 kit by producing two DNA sample-indexed libraries in a multiplexed PCR^11^.

### Statistical analysis

The analysis of our results was based on SPSS statistical software (version 24), the results are expressed in percentage (%) and in number of staff for the qualitative variables. We used the chi- square test for comparison between quantitative and qualitative variables. The difference was considered significant when the P <0.05.

## Results and discussion

### Distribution of CCR according to the mutational status of the KRAS gene

This retrospective anatomo-clinical study consists of 91 patients, showing that 46.2% (n = 42) of patients with CRC having a KRAS mutation and 53.8% (n = 49) with wild-type and non-mutated KRAS (figure 01).

**Figure 1:**
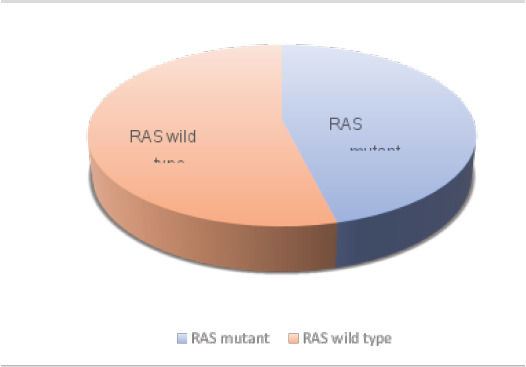
Distribution of CRC in patients according to the mutational status of the KRAS gene

This analysis shows that there is a significant difference (P≤0.0001) that has been demonstrated between patients with CRC and KRAS.

Over the past few decades, research into colorectal cancer (CRC) genomics and epigenetics has significantly advanced, revealing three major pathways driving carcinogenesis: chromosomal instability (CIN), microsatellite instability (MSI), and the CpG island methylator phenotype (CIMP) pathway^9^. Among these, CIN—characterized by whole-chromosome gains or losses is the most common, arising from cumulative mutations in oncogenes and tumor suppressor genes^17^. A critical early event in this process is KRAS mutation, which dysregulates oncogenic signaling pathways and promotes tumor progression^12^. Our study corroborates the pivotal role of KRAS in CRC, demonstrating its association with aggressive clinicopathological features in an Eastern Algerian cohort.

Consistent with prior studies, we found that KRAS mutations correlate with poor prognosis, aligning with evidence that these tumors exhibit enhanced proliferative capacity and resistance to therapy^29^. For instance, Barault et al. (2008) reported that KRAS-mutated CRCs are linked to reduced survival, while Conlin et al. (2005) observed significantly worse outcomes in patients harboring these mutations. Our findings further support this paradigm, as KRAS-altered tumors in our cohort displayed distinct anatomical localization and advanced staging, suggesting a more aggressive phenotype.

Recent work by Cremolini et al. (2023) highlights that KRAS G12C and non-G12C variants confer differential resistance to anti-EGFR therapies, underscoring the need for mutation- specific therapeutic strategies. Additionally, Vitiello et al. (2024) demonstrated that KRAS- mutated CRCs exploit immune evasion mechanisms, potentially explaining their poorer response to immunotherapy. These insights reinforce the clinical urgency of early KRAS profiling to guide treatment decisions.

In our cohort, no significant sex-based difference was observed in KRAS mutation prevalence (P=0.923), contrasting with a Moroccan study reporting a female predominance (50.75% vs. 49.24% males)^15^ This discrepancy may stem from regional genetic or environmental factors, such as dietary habits or consanguinity rates, which vary across North Africa^31^. Notably, Adjei et al. (2022) emphasized that African populations are underrepresented in CRC genomic studies, leading to gaps in understanding mutation distribution across demographics. Our study addresses this by providing Algerian-specific data, which may better inform local screening and therapeutic guidelines. Our study underscores the high prevalence and clinical significance of KRAS mutations in Algerian CRC ptients, reinforcing their role in tumor aggressiveness and therapeutic resistance. By integrating these findings with regional precision oncology initiatives, we can improve outcomes for this underserved population.

The study of western Algeria during the period 2000-2006 on a population that includes 501 patients with CRC, the results are 54.3% for men and 45.7% for women^16^.

### Distribution of CRC as a Function of Age and Mutational Status of the KRAS Gene

Our study shows that the age group most affected by CRC is that which is ≥ 41 years old with a mutational status of KRAS and a frequency of 40 and K wild RAS of 37cas, while patients whose age is lower than 40 and greater than 23 years are less affected by this disease whose frequency is respectively 4 in patients with KRAS mutated and 10 with wild KRAS.

According to the analyze, there is no significant difference (P = 0.107) that has been demonstrated between age and patient involvement by CRC with mutational status of the KRAS gene (Table 01).

**Table 1:** Distribution of subjects according to age and mutational status of the KRAS gene

	Gene KRAS mutant	KRAS wild-type	Total
Age ≤40	4	10	14
Age ≥41	40	37	77
Total	44	47	91

Age is a highly debatable prognostic factor, with six of 15 studies evaluating this factor concluding that the occurrence of CRC in an elderly patient was a factor in poor prognosis.

### Distribution according to the localization of the primary tumor

We found that the primary tumor in the majority of cases is at the rectum and sigmoid level. From our results we found 22 cases localized in the rectum (9 cases have a K RAS mutation and 13 with wild KRAS). Our analysis revealed that age remains a contentious prognostic factor in colorectal cancer (CRC). Among studies evaluating this parameter, 40% (6/15) identified advanced age as a predictor of poor outcomes. In our cohort, the majority of cases occurred in patients >50 years, aligning with global trends^36^. The debated role of age in CRC prognosis may reflect competing risks in elderly patients (e.g., comorbidities) rather than intrinsic tumor biology. Our findings align with Akdeniz et al. (2021), who reported that metastatic presentation at diagnosis more common in older adults drives poor outcomes independently of molecular status. Distribution of CRC by size of primary tumor and KRAS mutation

Our study shows that the majority of patients with CRC have a primary tumor size greater than 5cm including 21 cases (10 cases with mutated KRAS and 11 cases wild RAS). While this less than 5cm are 18 cases (11 cases KRAS mutated and 7 cases KRAS wild). The rectal/sigmoid predominance in our cohort contrasts with right-sided CRC trends in Western populations^2^, potentially due to regional dietary or genetic factors^33^. Left-sided tumors typically exhibit higher KRAS mutation frequencies^29^, yet our observed 41% mutation rate in rectal tumors suggests Algerian-specific patterns warranting further study.

### Distribution of CCR as a function of PTNM stage and KRAS gene mutation

Our study shows that stage IV is the most common among the stages of CRC with 28/50 (15 cases with a mutation at the KRAS gene level and 13 wild type KRAS), stage III and II in second position with 9 cases (III: 4 KRAS mutated and 5 wild-type KRAS, II: 6 cases KRAS mutated and 3 wild- type KRAS) and stage I presented only 4/50 cases (1 mutated KRAS and 3 wild-type KRAS). The high Stage IV prevalence (56%) underscores Algeria's late-diagnosis crisis, consistent with low- resource regions^36^. The 54% KRAS mutation rate in metastatic cases mirrors global data^34^, reinforcing KRAS's role in aggressive dissemination. Notably, Stages III/II showed balanced KRAS distribution, suggesting mutation-independent progression in locally advanced disease.

From our results we found that there is no significant difference (P = 0.399) in tumor size between mutated KRAS and wild-type KRAS (Table 02).

**Table 02:** Distribution according to tumor size and KRAS gene mutation

	Gene KRAS mutant	Gene KRAS wild-type	Total
**Size of tumor We** **2As ^2^<5**	11	7	18
**≥5**	10	11	21
**Total**	21	18	39

In contrast, they found that patients in southern Tunisia with CRC had a correlation (P = 0.036) between the KRAS mutation and tumor size when it was> 5 cm. At the same time, there is no significant difference (P = 0.356) between the PTNM stage and the RAS mutation (Table 03).

**Table 3:** Distribution according Stade PTNM and RAS mutation

		Gene KRAS mutant	Gene KRAS wild-type	Total
	I	1	3	**4**
	II	6	3	**9**
**Stade P TNM**	III	4	5	**9**
	IV	16	13	**28**
**Total**		**26**	**24**	**50**

The clinical status of our findings is inconsistent with literature studies in which they show that the sum of stage III and IV patients is less common than stage I and stage II^18^.

CCR distribution according to the number of lymph node metastases with mutational status of KRAS gene In our study the number of patients with lymph node metastases is 48/76 cases (17 with mutated KRAS and 31 wild KRAS).

This analysis shows that there is a significant difference (P = 0.015, P≤0.05) between the presence or absence of lymph node metastases in patients with CRC and the KRAS mutation (Figure 03).

**Figure 3:**
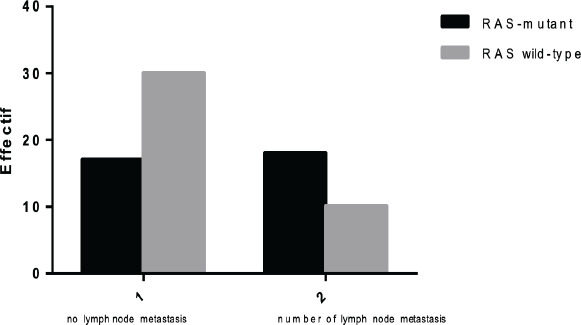
Distribution of CCR according to the number of lymph node metastases with mutational status of KRAS gene

Tumors exceeding the muscularis (T3), directly invading neighborhood structures (T4) or with lymph node metastases or at a distance are poor prognostic factors^20^. As the degree of differentiation decreases, the frequency of lymph node metastases increases and survival decreases.

The Damak et al. (2015)^17^ study is in agreement with us or patients in southern Tunisia with CRC, including lymph node involvement (p = 0.032).

### Metastatic extension

We noted in our series that the total number of patients attaining CRC is 64 cases, the maximum damage is liver metastasis reaches 26 cases (11 cases has a KRAS mutation), while pulmonary and peritoneal metastases including the total of cases is 13 (8 cases with mutated KRAS and 5 cases with wild-type KRAS), and in umbilical and adrenal and muscle and appendix metastases is 1 case with mutated KRAS in each type. In the Mediastinal with peritoneal and ovarian carcinomatosis and intestine and recto-peritoneal metastasis also only 1 case with wild KRAS in all patients.

The observed metastatic patterns in our cohort of 64 CRC patients demonstrate several clinically relevant trends. Liver metastases were by far the most common (40.6% of cases), which aligns with the established understanding of colorectal cancer's preferential hematogenous spread through the portal venous system^28^. Interestingly, we found no significant association between KRAS mutation status and liver metastasis development (P=0.462), suggesting that other biological factors may be more influential in determining hepatic tropism^30^. This finding contrasts with the pulmonary and peritoneal metastases, where we observed a trend toward higher KRAS mutation prevalence (61.5%), although this did not reach statistical significance in our cohort. This pattern may reflect the known role of KRAS mutations in promoting more invasive phenotypes through epithelial-mesenchymal transition and enhanced migratory capacity^29^,^31^.

The rare metastatic cases in our study (affecting umbilical, adrenal, muscle and appendix sites) all occurred in KRAS-mutated tumors, which could indicate either a stochastic effect in our limited sample or potentially a true bological propensity of KRAS-driven tumors to spread to these unusual sites. The wild-type KRAS cases that metastasized to mediastinal, ovarian and recto- peritoneal locations suggest that alternative molecular pathways may facilitate spread to these particular sites^33^.

From a clinical perspective, these findings have several important implications. First, the high frequency of liver metastases reinforces the need for regular hepatic surveillance in CRC patients. Second, the trend toward KRAS mutation enrichment in pulmonary and peritoneal metastases may have therapeutic implications, as these patients would be less likely to benefit from anti-EGFR therapies^34^. The presence of rare metastases, while uncommon, reminds clinicians to maintain a high index of suspicion for unusual presentations in advanced CRC cases^32^.

There was no significant difference (P = 0.462) that was demonstrated between the metastatic organs and the mutation in the KRAS gene and the involvement of patients by CRC (table 04)

**Table 4:** Distribution of CCR by metastatic organ and KRAS mutation

	gene			Total
	KRAS-mutant	KRAS Wild-type		
Metastatic organs	Liver	11	15	26
	Pulmonary	8	5	13
	peritoneal	8	5	13
	bone	1	1	2
	Umbilical	1	0	1
	ovarian	0	1	1
	Mediastinal with peritoneal carcinomatosis	0	1	1
	adrenal	1	0	1
	Intestinal	0	1	1
	Muscular	1	0	1
	Double-peritoneal	0	1	1
	Duodenum	2	0	2
	appendix	1	0	1
Total		34	30	64

Mutations in KRAS often result in constitutive activation of signaling pathways that promote growth, survival, tumor progression, metastasis, and angiogenesis^18^.

A number of previous studies have reported a high degree of concordance in the mutational status of KRAS between primary tumors and liver metastases. The liver and lungs are common sites of CRC metastases^12^.

### Distribution of KRAS mutations and their frequency in the CCR

According to our analysis results, the KRAS mutation was detected at the level of 3 exons (2 and 3 and 4), most of the mutations are present in exon 2 which consists of five mutations (P.G13D (C.38G> A), P.G12A (C.35G> C), P.G12C (C.34G> T), P.G12D (C.35G> A), P.G12V (C.35G>T); )) the most common of which is P.G12D (C.35G> A) of 10 cases, exon 3 to 2 mutation type (P.Q61K (C.181C> A) and P.Q61L (C.182C> A) )) represents in each mutation 1 single case in each mutation, whereas at exon 4 there is a single mutation (P.A146T (C.436G> A)) (Table 05).

**Table 5:** Distribution of KRAS mutations and their frequency in the CCR

Codons										
	P.G13D (C.38G>A)		P.G12A (C.35G>C)	P.G12C (C.34G>T)	P.G12D (C.35G>A)	P.G12V (C.35G>T)	P.Q61K (C.181C>A)	P.Q61L (C.182C>A)	P.A146T (C.436G>A)	Total
Exons	exon 2	7	5	5	10	3	0	0	0	30
	exon 3	0	0	0	0	0	1	1	0	2
	exon 4	0	0	0	0	0	0	0	1	1
Total		7	5	5	10	3	1	1	1	33

Of the 65 tumors containing mutated KRAS genes, 3 had unique mutation patterns. He found three types of mutation: a homozygous mutation (C12GGT> TGT with amino acid change from Gly to Cys and C13GGC> GAC with amino acid change from Gly to Asp) and the third case with 2 mutated nucleotides in the codon 12 may represent a double mutation (GGT> TTT with amino acid change from Gly to Lys) or 2 different mutations in two alleles (GGT> TGT and GGT> GTT with amino acids go from Gly to Cys and Gly to Val, respectively).

The most common types of mutations observed in KRAS in all human cancers are the G> A transition and the G> T transversion. Although the precise molecular and cellular mechanisms that constitute the oncogenic effects of KRAS mutation activation remain poorly understood^18^.

The results of Damak et al., (2015)^17^ agree with us; they showed that the sequencing of the PCR products made it possible to detect the presence of two mutations in the heterozygous state: at codon 12: p. G12D (c.35G> A) and codon 13: p.G13D (c.38G> A)^19^.

The KRAS gene encodes a small 21 kDa protein that is transiently activated in response to extracellular stimuli or signals such as growth factors, cytokines, and hormones via cell surface receptors^18^.

Upon activation, the KRAS protein is also able to: deactivate the signaling pathway by catalyzing the hydrolysis of guanosine triphosphates (GTPs) to guanosine diphosphates. The most common KRAS mutations in codons 12 and 13 are activation mutations, leading to continuous activation of downstream pathways^23^.

Other mutations are generally observed at codons 61, 59, 117 and 146 (less than 10% of cases)^24^. Confirmation of the absence of somatic mutations at codons 12 and 13 of the KRAS gene is a prerequisite for patients with advanced chemorespecific CRC to benefit from anti-EGFR Therapy^25^.

The vast majority of KRAS mutations is localized in codons 12 and 13, and remain in codons 61, 146 and other residues 26,37. While mutations 12 and 13 of the point KRAS neuralgic do not match interfere with its ability to associate with GAPs they alter the position of a residue.

## Conclusion

This study highlights the high prevalence of KRAS mutations in colorectal cancer (CRC) within an Eastern Algerian cohort, reinforcing its role as a key molecular driver of aggressive disease. Our findings demonstrate that KRAS-mutated tumors exhibit distinct clinicopathological features, including preferential anatomical localization, advanced staging at diagnosis, and a unique co-mutation profile, suggesting an underlying biological divergence from wild-type counterparts.

The association between KRAS mutations and more aggressive tumor behavior underscores the critical need for early molecular profiling in CRC management. Given that KRAS alterations confer resistance to anti-EGFR therapies, their detection is not only prognostic but also essential for guiding first-line treatment decisions in metastatic disease. Furthermore, the observed mutation patterns in this Algerian cohort may reflect population-specific genetic or environmental influences, warranting further investigation into regional carcinogenic drivers.

Despite these insights, significant challenges remain in effectively targeting KRAS-driven CRC. While the development of direct KRAS inhibitors (e.g., sotorasib, adagrasib) has revolutionized treatment for KRAS-mutated lung cancer, their efficacy in CRC remains limited, likely due to tumor microenvironment differences and compensatory pathway activation. This emphasizes the need for novel therapeutic strategies, such as combination therapies targeting downstream effectors (e.g., MEK, ERK) or immune checkpoint modulation in microsatellite-stable tumors.

**Our perspectives are**

Larger, multi-center validation studies to confirm the generalizability of our findings across North African populations.
Comprehensive genomic profiling to identify co-occurring alterations (e.g., TP53, PIK3CA) that may influence therapeutic response.
Functional studies to elucidate the mechanistic basis of KRAS-associated aggressiveness in CRC. Clinical trials evaluating KRAS-targeted therapies in CRC, particularly in underrepresented populations like Algeria.
By integrating molecular diagnostics into routine practice and advancing precision oncology initiatives, we can ultimately improve outcomes for patients with KRAS-mutated CRC a subgroup historically associated with poorer survival and limited therapeutic options.

## Figures and Tables

**Figure 2:**
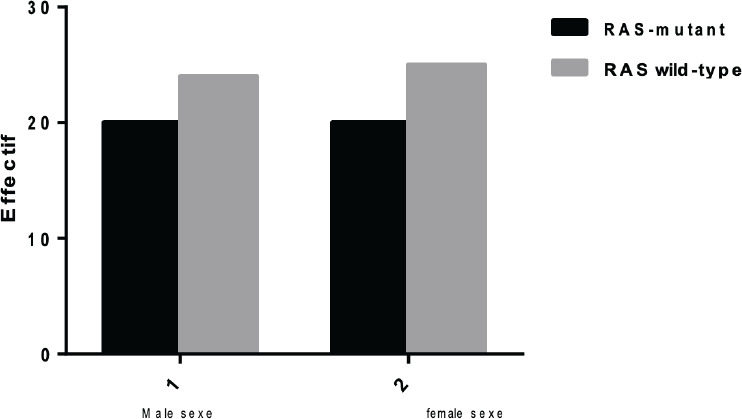
Distribution of CRC by sex and mutation or not of KRAS gene
